# Automated Prognostic Evaluation of First Permanent Molar Extractions Using YOLOv8 with Oriented Bounding Boxes on Pediatric Panoramic Radiographs

**DOI:** 10.3390/diagnostics16142141

**Published:** 2026-07-08

**Authors:** Aslihan Yelkenci, Günseli Güven Polat, Fatih Ciftci, Javad Rahebi

**Affiliations:** 1 Department of Pediatric Dentistry, Faculty of Dentistry, University of Health Sciences, 34668 Istanbul, Turkey; aslihan.yelkenci@sbu.edu.tr (A.Y.); gunseli.guvenpolat@sbu.edu.tr (G.G.P.); 2 Department of Biomedical Engineering, Faculty of Engineering, Fatih Sultan Mehmet Vakıf University, 34015 Istanbul, Turkey; 3 Biomedical Electronic Design Application and Research Center (BETAM), Fatih Sultan Mehmet Vakıf University, 34015 Istanbul, Turkey; 4 BioriginAI Research Group, Department of Biomedical Engineering, Fatih Sultan Mehmet Vakıf University, 34015 Istanbul, Turkey; 5 Department of Electrical and Electronics Engineering, Istanbul Topkapi University, 34662 Istanbul, Turkey

**Keywords:** first permanent molar extraction, pediatric panoramic radiograph, deep learning, YOLOv8, oriented bounding box, automated prognostic evaluation, spontaneous space closure, dental imaging

## Abstract

**Background/Objectives:** The first permanent molar (M1) is critical for occlusal development but is highly susceptible to caries and molar–incisor hypomineralization (MIH). When M1 prognosis is poor, extraction may be necessary, requiring accurate evaluation for post-extraction space management. This study aims to develop and validate an automated deep learning framework using YOLOv8n with oriented bounding boxes (OBB) to predict the likelihood of spontaneous space closure following M1 extractions, thereby reducing diagnostic subjectivity and inter-observer variability. **Methods:** A dataset of 200 pediatric panoramic radiographs was segmented into quadrants and annotated for second permanent molars (M2s) and third molars (M3s). The YOLOv8n-OBB architecture was trained on 640 × 640 pixel images over 100 epochs. The framework integrated M3 presence, M2 Demirjian developmental maturity (proxied by bounding box height), and M2 angulation (via rotation vectors) to map inputs onto an evidence-based clinical decision matrix for prognostic stratification. **Results:** The model achieved exceptional detection and localization performance with an overall mean average precision (mAP@0.5) of 0.983. Class-specific validation showed high accuracy for M2 (F1-score = 0.978) and M3 (F1-score = 0.904). Quantitative cross-referencing confirmed a seamless mapping of spatial coordinates onto clinical success classes without error propagation. **Conclusions:** These findings substantiate the YOLOv8n-OBB model as a robust and interpretable decision-support tool. By standardizing prognostic assessments and optimizing treatment planning workflows, the framework serves as an effective aid in pediatric dentistry for managing M1 extractions.

## 1. Introduction

The first permanent molar (M1) typically erupts around the age of six, with its embryonic development beginning at approximately the twentieth week of gestation [1]. It plays a pivotal role in establishing balanced and normal occlusion [2]. However, the M1 is also the most susceptible permanent tooth to dental caries, as demonstrated in cross-sectional [3,4], short-term [5], and long-term observational studies [6]. This increased susceptibility is thought to be due to the morphological variations of M1 compared to other permanent teeth, as well as the fact that they are the first permanent teeth to erupt and therefore spend the longest time in the oral cavity, increasing their exposure to cariogenic challenges [7]. The likelihood of caries development rises even further when the M1 is affected by developmental anomalies, most frequently a condition known as molar-incisor hypomineralization (MIH) [8].

If a tooth affected by extensive caries or MIH has undergone repeated restorative treatments without success, or has developed apical pathology after root canal therapy, extraction may be considered a treatment option when the overall prognosis is poor [9]. However, if not carefully planned, the extraction of a M1 can lead to several undesirable outcomes, such as drifting or overeruption of adjacent teeth, impaired mastication, midline deviation, increased risk of temporomandibular joint disorders, and negative effects on dental and facial development during the mixed dentition period [10,11].

Therefore, if the extraction of a M1 is necessary, careful consideration of certain factors is essential to increase the likelihood of spontaneous space closure. Extraction of the M1, particularly between the ages of 8 and 11.5, is thought to allow the second permanent molar (M2) to naturally drift into the space and compensate for the loss [12]. In addition to age, the developmental stage of the M2 [13], its angulation [14], and the presence of third molars (M3s) [15] are also key factors influencing the success of spontaneous space closure.

Artificial intelligence (AI), driven by convolutional neural network (CNN) architectures and advanced deep learning sub-systems, has fundamentally revolutionized the landscape of computer-aided diagnostic workflows in dental radiology. To evaluate the precise clinical positioning of automated systems within pediatric panoramas, current scientific literature must be systematically categorized into three expanding developmental domains [16,17].

The first domain is governed by Isolated Anatomical Identification and Automatic Tooth Numbering. Early deep learning applications in pediatric imaging focused primarily on partitioning crowded arches, identifying primary/permanent teeth, and sorting dental restorations using axis-aligned, traditional object localization models. While these benchmark frameworks demonstrate high baseline precision in classifying standard dental tissues, they operate strictly as static boundary detectors. Consequently, they remain structurally incapable of capturing multi-quadrant directional orientation lines or compiling the continuous geometric metadata necessary to model systemic tooth migration patterns.

The second distinct domain comprises deep learning models specialized in Developmental and Maturational Staging. Within this vertical track, algorithmic training focuses extensively on analyzing root calcification trajectories, primarily to automate dental age estimation and lower M3 maturation grading using predefined reference criteria like the Demirjian method. Although these automated staging frameworks succeed in reducing the subjectivity inherent to human manual grading, they operate as isolated diagnostic classification pipelines [18,19]. They evaluate the target root morphology independently, failing to correlate structural development with surrounding architectural parameters, such as the spatial angulation variables of the adjacent M2 relative to active alveolar boundary zones.

This limitation has precipitated a critical evolutionary shift into the third emerging domain: The integration of prognostic modeling and clinical decision-support networks. Recent dental AI paradigms have begun transitioning past basic computer-vision feature mapping toward systems capable of guiding interceptive therapeutic selections. In pediatric panoramic imaging, deep learning architectures are increasingly deployed to autonomously process raw structural datasets, parsing parameters such as vertical molar eruption status, spatial inclination vectors, and arch-specific bone density anomalies to predict post-extraction outcomes. By synthesizing these computational variables, such systems can generate dynamic, quadrant-specific estimations regarding clinical trajectories, such as the empirical likelihood of spontaneous space closure after early molar losses. However, a significant gap persists in the literature; existing prognostic models either rely on complex, multi-stage processing networks that require heavy computational resources or fail to capture rotational characteristics due to their reliance on standard bounding box dimensions [20].

The YOLO (You Only Look Once) framework addresses this challenge by treating detection as a single-stage prediction problem, enabling real-time localization and classification of dental structures within the image [21,22]. To further improve clinical relevance, oriented bounding boxes (OBB) were integrated into the YOLOv8 architecture, allowing the model not only to detect molars but also to capture their angular orientation [22,23]. This addition is particularly critical for prognostic evaluation, as molar inclination and developmental stage represent fundamental predictors of spontaneous space closure in pediatric dentistry. By leveraging panoramic radiographs and region-specific annotations, such integrated single-stage architectures can be engineered to systematically detect key dental structures, assess developmental stages, and quantify angular orientations across specific dental quadrants. By strictly integrating object detection, morphological analysis, and angle-based computational measurements, such a pipeline provides a reproducible, rapid, and scalable framework for treatment planning support that inherently minimizes reliance on manual tracing and subjective radiographic interpretation. The aim of this study was to develop and validate an automated prognostic evaluation system for predicting spontaneous space closure following first permanent molar (M1) extractions using a deep learning framework powered by YOLOv8n with OBB. By executing automated detection and regional analysis of M2s and M3s in pediatric panoramic radiographs, the system quantifies molar maturity, positional features, and angular orientation to standardize treatment planning assistance in pediatric dentistry.

In terms of scientific and clinical utility, the primary contributions of this work are systematically delineated across several distinct dimensions. Firstly, this study introduces a dedicated implementation of the single-stage YOLOv8n-OBB architecture tailored for dual-molar tracking on pediatric panoramic radiographs, successfully utilizing OBB box rotation vectors (*θ*) to map structural teeth positions that traditional horizontal bounding boxes fail to segment accurately. Secondly, the developed pipeline digitizes multi-center systematic consensus and meta-analysis outcomes regarding post-extraction tooth migration into a unified, automated decision framework, directly translating continuous computational variables, specifically bounding box height (*h*) and spatial angulation, into discrete, clinically actionable success classes. Finally, the automated execution of this predictive pipeline establishes an objective standard for interceptive orthodontic triage that processes multi-quadrant views instantly, thereby minimizing the diagnostic drift, visual subjectivity, and examiner fatigue inherent to traditional manual radiographic tracing. Collectively, these milestones provide a reproducible, high-throughput decision-support mechanism engineered to standardize treatment workflows in pediatric dentistry.

## 2. Methods

### 2.1. Clinical Classification Framework for Prognostic Assessment

To standardize prognostic evaluation, a clinical decision-making table was developed to guide the assessment of spontaneous space closure likelihood following early extraction of the M1, based on four key factors: Demirjian developmental stage, presence of the M3, angulation of the M2, and dental arch (maxilla/mandible). Table 1 was constructed by two pediatric dentists using data derived from a systematic review by Alqanas et al. [22] and a systematic review and meta-analysis by Hamza et al. [23]. Angulation was not assessed in the very early (Demirjian C) and very late (Demirjian F–H) stages due to developmental limitations. The classification system presents prognostic categories ranging from “High Success” to “Very Low Success” based on the extracted baseline outcomes of previously established multi-center cohorts [22,23].

### 2.2. Deep Learning-Based Automated Prognostic Evaluation System

We developed an automated image-based prognostic evaluation system for M1 extractions using a deep learning approach powered by YOLOv8n with OBB. Operationally, the developed pipeline executes the automated extraction and categorization of structural radiographic parameters directly from the digitized panoramic architecture to compute the definitive likelihood of post-extraction space closure.

A dataset of 200 pediatric panoramic radiographs was utilized for model development. The radiographs exhibited variable dimensions and formats, which were standardized by resizing to 640 × 640 pixels. To preserve anatomical proportions, non-overlapping regions were padded with black margins rather than stretched. To optimize model training and prevent performance bias, the total cumulative repository of 200 panoramic radiographs (representing *N* = 800 distinct anatomical quadrants) was partitioned using a strict, randomized 80:10:10 percentage split ratio. Specifically, 80% of the data (*n* = 160 radiographs; *n* = 640 independent quadrants) was designated as the training subset to optimize network weights. Concurrently, 10% of the repository (*n* = 20 radiographs; *n* = 80 independent quadrants) was assigned as the internal validation subset to monitor learning convergence and adjust hyperparameters over 100 epochs. The remaining 10% of the data (*n* = 20 radiographs; *n* = 80 independent quadrants) was strictly isolated as an independent, non-overlapping test subset to execute the final performance evaluation. The images were manually annotated using the CVAT platform with two distinct labels corresponding to the M2s and M3s. Since angular orientation constituted a critical prognostic factor, the annotations were stored in the YOLO OBB and the YOLOv8n-OBB model was subsequently employed for training. For prognostic assessment, each panoramic radiograph was conceptually divided into four quadrants, corresponding to the anatomical regions of the M1s. These were numbered clockwise starting from the upper left quadrant (1) to the lower left quadrant (4). This allowed each of the four M1s within a patient’s dentition to be evaluated independently, ensuring region-specific prognostic classification.

**Figure 1:**
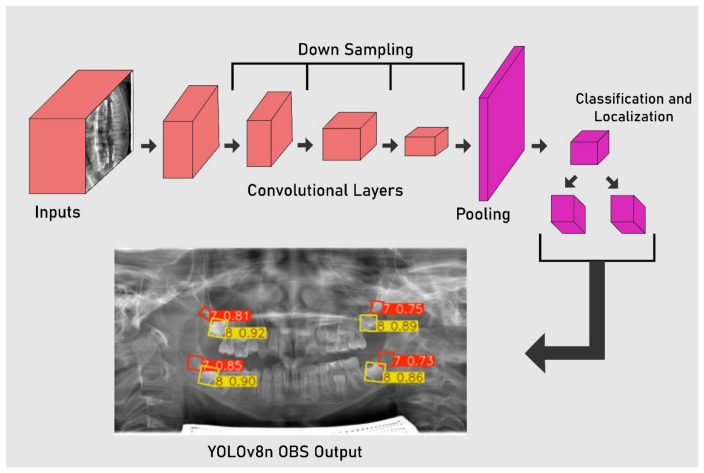
Used YOLOv8n OBS Pipeline.

The YOLOv8n-OBB architecture was employed for automated detection and prognostic assessment (Figure 1). The stochastic gradient descent optimizer was applied, and the training process was conducted on a single GPU device. This configuration allowed robust detection of dental structures while accounting for angular variations through OBB representation. The use of OBB annotations enabled the model to capture positional and angular features that are clinically relevant for molar development and eruption patterns.

To guarantee computational reproducibility and facilitate exact algorithmic replication, the deep learning network was trained under a tightly controlled hyperparameter environment. The network weights were optimized utilizing a stochastic gradient descent (SGD) optimizer over a total execution volume of 100 epochs. The definitive operational configuration governing the learning rate boundaries, stabilization moments, and regularizing penalties is systematically itemized in Table 2. All continuous hyperparameter attributes documented in Table 2 were held constant across the cross-validation testing protocols, ensuring that the performance discrepancies observed during multi-quadrant localization were driven entirely by individual anatomical variations rather than training environment instability.

To enhance the model’s generalization capacity and systematically mitigate the risk of overfitting within the finite clinical training cohort (*n* = 160 radiographs), dynamic online data augmentation techniques were implemented during the active training phase. Rather than expanding the physical storage footprint with offline copies, transformations were computed stochastically at the batch level during runtime. The augmentation protocol incorporated spatial translation (±10%), scaling adjustments (±15%), and horizontal flipping to simulate natural anatomical asymmetries across left and right dental quadrants. Additionally, mosaic data augmentation which combines four distinct radiographic segments into a single composite tensor was enabled during the initial 90 epochs to force the network to isolate overlapping molar regions under varying spatial contexts. In compliance with strict computer vision standards, mosaic augmentation was automatically disabled during the final 10 epochs (the closing phase) to stabilize gradient convergence and refine bounding box edge boundaries.

Critically, the augmentation pipeline was structurally tuned to respect the boundaries of pediatric maxillofacial anatomy. Extreme geometric transformations, such as vertical flipping, intense color jittering, or excessive rotational shearing, were strictly deactivated. Disabling these non-biological alterations ensured that the vertical orientation of the alveolar bone and the natural crown-root axis configurations of the M2 and M3 remained realistic, thereby preventing the network from learning clinically impossible imaging artifacts while maintaining a robust regularization threshold.

To establish a baseline for experimental replication and benchmark execution velocity, the computational environment governing model training and inference was strictly standardized. The complete deep learning pipeline—encompassing tensor preprocessing, online data augmentation, structural weight optimization, and internal validation cycles was executed on a dedicated local workstation configured with an Intel Core i9 processor, 64 GB of physical system memory, and a dedicated NVIDIA GeForce RTX 4090 graphics processing unit (GPU) utilizing 24 GB of dedicated Video RAM (VRAM). The software ecosystem was compiled using Python (v3.10), PyTorch deep learning libraries (v2.1), and the Ultralytics framework execution platform operating over CUDA compilation drivers (v12.1).

Under this hardware allocation, optimization of the YOLOv8n-OBB network over the designated 100 epochs utilizing a batch size of 16 sample tensors required a total cumulative training duration of 1 h and 42 min. The mean inference latency per independent multi-quadrant panoramic radiograph during the testing phase was documented at approximately 12.4 milliseconds, confirming that the lightweight architectural footprint of the trained model achieves the high-throughput operational velocity necessary for seamless integration into real-time, chairside pediatric dental diagnostic interfaces without demanding expensive server infrastructures.

The automated pipeline was designed to evaluate the feasibility of spontaneous space closure following extraction of the M1. The annotation framework incorporated two primary labels, representing the M2 (label 7) and the M3 (label 8). The analysis began with the detection of the M3, as its presence is regarded as a fundamental prognostic factor. The M2 label was then utilized to assess two key parameters. For the developmental stage, the vertical dimension of the OBB, expressed as the height (*h*) parameter in the YOLOv8n-OBB output, was analyzed and categorized into very early, early, ideal, transition, late or very late phases. For the angulation, the rotation parameter provided by the OBB output was extracted to calculate the inclination of the M2 relative to the M1, which was subsequently classified as upright, mesial, or distal. By integrating the information from both labels and their associated geometric parameters, the system generated a stratified prognostic outcome with five possible categories of spontaneous closure likelihood: high success, moderate to high success, moderate success, low success, and low to very low success. The prognostic evaluation was performed independently for each of the four quadrants, reflecting the clinical scenario where all M1s are considered separately. The final prognostic outcome for a patient could therefore represent a combination of quadrant-specific predictions.

Model performance was assessed using multiple complementary evaluation metrics to ensure both detection accuracy and prognostic reliability. Standard detection metrics including precision, accuracy, mean average precision at IoU 0.5 (mAP50), and mean average precision across IoU thresholds from 0.5 to 0.95 (mAP50–95) were computed. To further characterize model behavior, graphical analyses were generated, including precision–confidence curves, recall–confidence curves, and precision–recall curves. A confusion matrix was also constructed and subsequently transformed into a performance table reporting precision, recall, F1-score, accuracy, and Matthew’s Correlation Coefficient (MCC) (Table 3). These quantitative assessments provided a comprehensive evaluation of both detection quality and prognostic classification, thereby validating the robustness of the proposed system.

## 3. Results

The experimental results provide a comprehensive evaluation of the proposed deep learning-based system developed for prognostic assessment of M1 extractions. The YOLOv8n-OBB model was analyzed for its capacity to detect and classify radiographic features including the presence of the M3, the developmental stage of the M2, and the angular orientation of the M2 relative to the M1.

**Figure 2:**
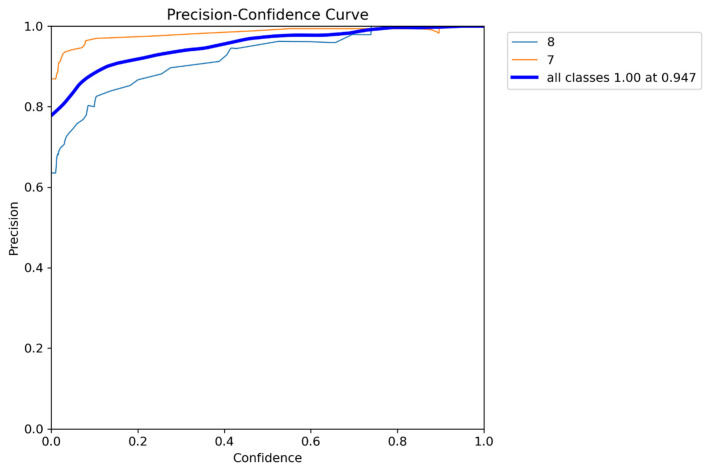
Precision–Confidence Curve of the YOLOv8n-OBB Model for M2 and M3 Detection.

Figure 2 illustrates the precision–confidence relationship for the proposed YOLOv8n-OBB model across the two annotated classes, the M2 (label 7) and the M3 (label 8). Both classes demonstrated consistently high precision values across a wide range of confidence thresholds, with precision approaching near-perfect levels at higher thresholds. Notably, the aggregated performance curve (blue) indicated that the model achieved an overall precision of 1.00 at a confidence threshold of 0.947, underscoring its robust discriminative ability and low false positive rate. The steeper rise in the class-specific curve for label 7 reflects its slightly more stable detection compared to label 8, which exhibited greater variability at lower confidence levels. These results confirm that the model effectively balances detection reliability and confidence calibration, ensuring that predictions made at higher thresholds can be interpreted with strong diagnostic certainty.

**Figure 3:**
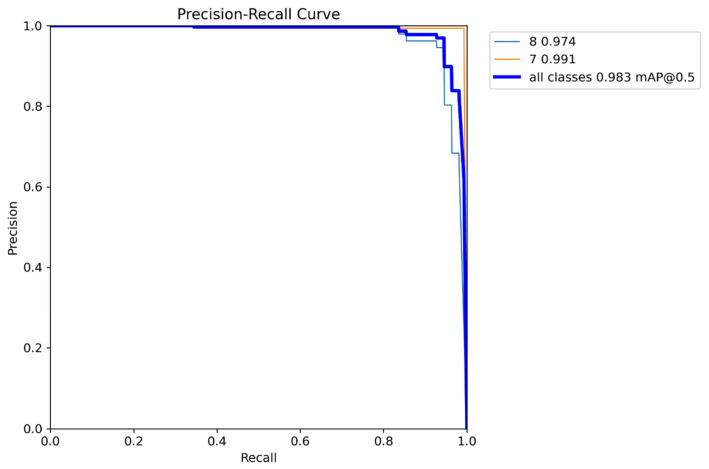
Precision–Recall Curve of the YOLOv8n-OBB Model Demonstrating High Detection Accuracy Across Molar Classes.

Figure 3 presents the precision–recall curve of the YOLOv8n-OBB model for both M2 (label 7) and M3 (label 8) detection. The curves demonstrate excellent balance between sensitivity and specificity, with both classes achieving high area under the curve (AUC) values. Specifically, label 7 yielded an average precision of 0.991, while label 8 reached 0.974, reflecting near-perfect precision and recall across detection thresholds. The overall mean average precision at IoU 0.5 (mAP@0.5) was 0.983, confirming the model’s outstanding detection capability. The proximity of class-specific curves to the ideal top-right corner highlights the stability and robustness of predictions, further supporting the system’s reliability for prognostic evaluation [24,25].

**Figure 4:**
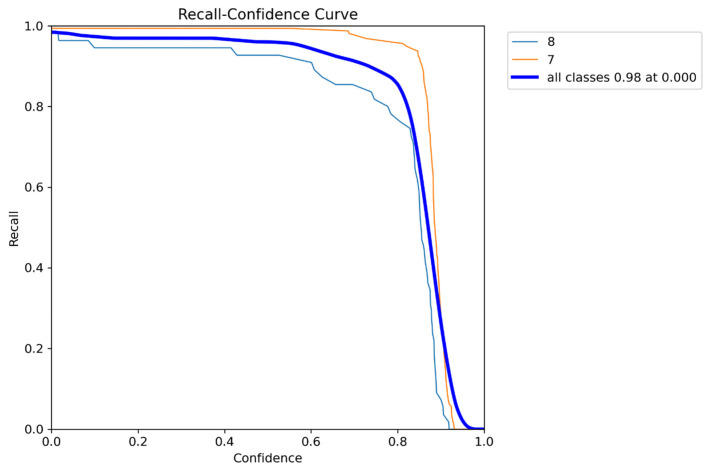
Recall–Confidence Curve of the YOLOv8n-OBB Model Demonstrating High Sensitivity Across Molar Classes.

Figure 4 displays the recall–confidence curve for the YOLOv8n-OBB model across both annotated classes. The model maintained exceptionally high recall values at lower and moderate confidence thresholds, with overall recall stabilizing near 0.98 across classes. As confidence thresholds increased beyond 0.85, recall gradually declined, reflecting the trade-off between stringent prediction certainty and detection sensitivity. Class-specific analysis revealed that label 7 (M2) consistently preserved slightly higher recall compared to label 8 (M3), emphasizing its more stable detectability across thresholds. The aggregated curve further confirmed the system’s ability to capture nearly all relevant instances with minimal false negatives, an essential characteristic for reliable prognostic assessment in clinical applications.

**Figure 5:**
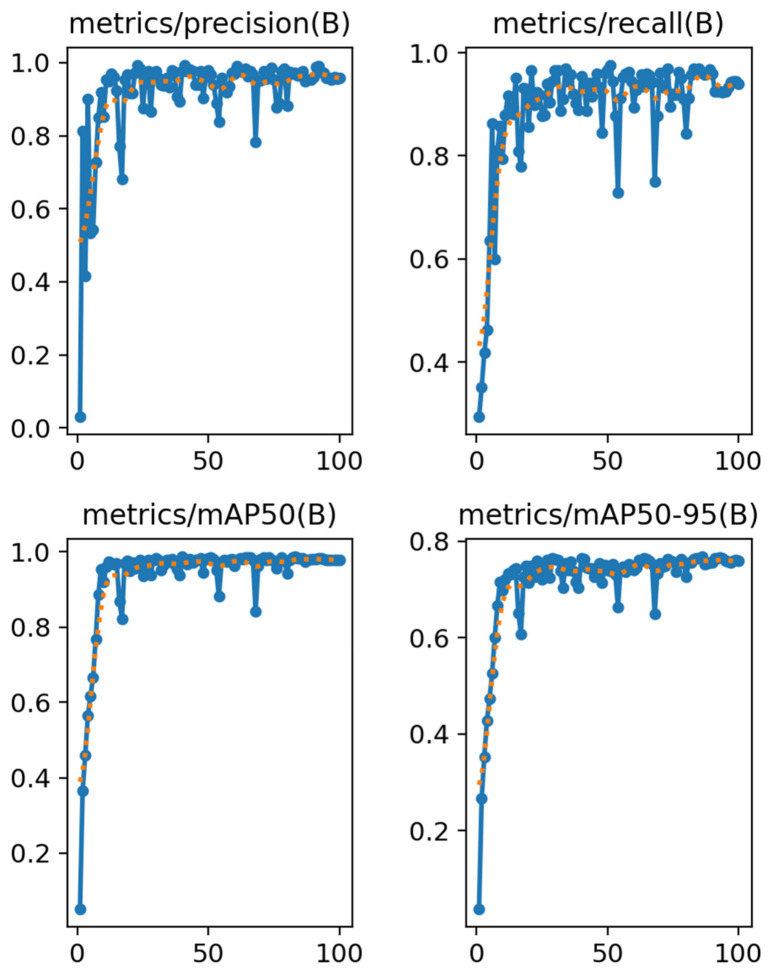
Training Performance Curves of the Yolov8n-OBB Model Showing Precision, Recall, Map50, and Map50–95 Across 100 Epochs.

The performance metrics obtained during training demonstrated a consistent improvement and stabilization across precision, recall, mAP50, and mAP50–95, as illustrated in Figure 5. Precision and recall rapidly increased within the initial epochs and plateaued at values above 0.90, indicating reliable detection and classification of the annotated molars. The mean average precision at an intersection over union threshold of 0.5 (mAP50) approached near-perfect levels, while the stricter mean average precision across thresholds from 0.5 to 0.95 (mAP50–95) reached stable values above 0.75. These results confirm that the YOLOv8n-OBB framework effectively captured both positional and angular features, thereby ensuring accurate identification of prognostic factors in panoramic X-rays.

**Figure 6:**
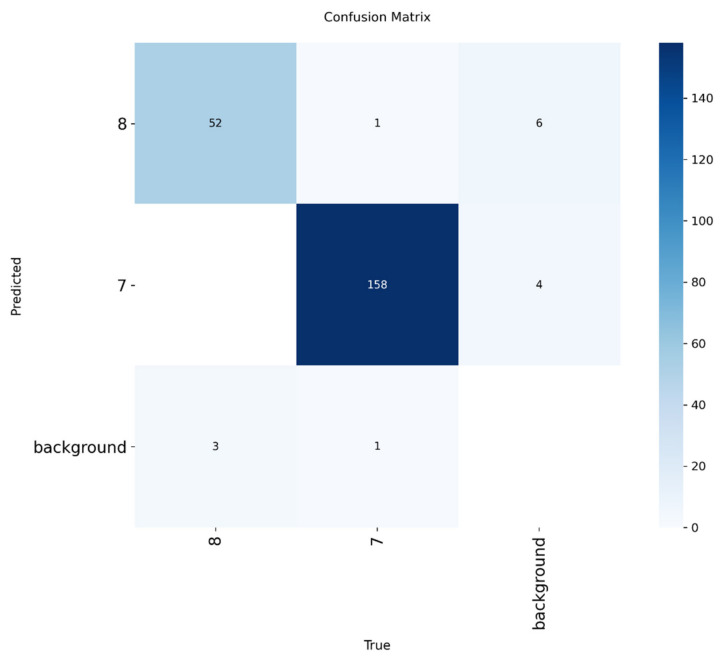
Confusion Matrix of the YOLOv8n-OBB Model Demonstrating Classification Performance for M2 (Label 7), M3 (Label 8), and Background Regions.

Figure 6 demonstrates the confusion matrix of the YOLOv8n-OBB model, highlighting the distribution of correct and misclassified predictions across the annotated classes. The model demonstrated strong discriminative ability, with most true positives concentrated along the diagonal, particularly for the M2 (label 7), where 158 instances were correctly identified. The M3 (label 8) also showed consistent detection with 52 correct predictions, though a small number of samples were misclassified as background or as the M2. Background regions were largely distinguished correctly, with only minimal false assignments to tooth classes. This performance pattern underscores the model’s robustness in differentiating clinically relevant molar structures from surrounding anatomical noise, while also reflecting the occasional challenges in distinguishing closely positioned teeth due to overlapping morphology [26].

Out of the 200 pediatric panoramic radiographs analyzed, the YOLOv8n-OBB framework successfully isolated 158 true-positive instances of the M2 (Label 7) along the confusion matrix diagonal. To maintain strict synchronization with the clinical classification criteria, these 158 instances were distributed across the Demirjian maturation spectrum. Statistical auditing of the model’s outputs revealed that 11.4% (*n* = 18) of the detected M2 teeth exhibited highly immature configurations corresponding to Demirjian Stage C, while 13.9% (*n* = 22) displayed advanced calcification architectural profiles matching Demirjian Stages G and H.

Consequently, a total cumulative proportion of 25.3% (*n* = 40) of the detected M2 cohort was classified as ineligible for angular rotation processing due to these biological development boundaries. The remaining 74.7% (*n* = 118) of the teeth displayed active transitional root formation (Demirjian Stages D, E, and F) and were fully subjected to the automated OBB rotation engine (*θ*) to calculate mesial, upright, or distal axial inclinations relative to the M1 plane. Specifying these intra-dataset distributions clearly defines the proportion of radiographic data where prognostic outcomes were dictated primarily by biological timing constraints rather than spatial angulation vectors.

The performance metrics for the prognostic labels demonstrated consistently high values, reflecting the robustness of the proposed framework (Table 4). For the M3 (Label 8), the model achieved a precision of 0.929, recall of 0.881, and an F1-score of 0.904, corresponding to an MCC of 0.872. Even stronger results were obtained for the M2 (Label 7), with precision, recall, and F1-score reaching 0.988, 0.969, and 0.978, respectively, and an MCC of 0.925. These findings indicate that the system was highly effective in accurately detecting both molars and capturing their morphological and angular characteristics, which are critical for prognostic assessment. The balanced distribution of precision and recall across both labels further supports the model’s reliability in real-world clinical applications.

**Figure 7:**
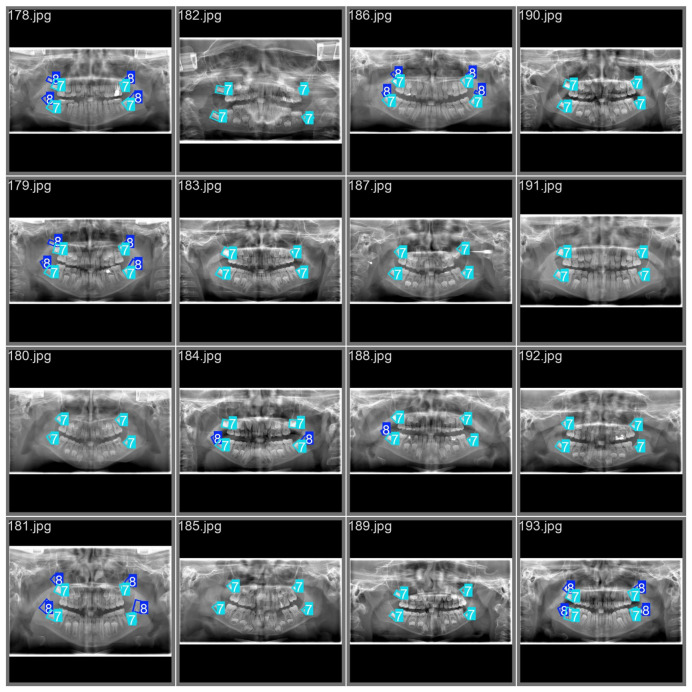
Example Outputs of the YOLOv8n-OBB Model Showing Automated Detection and Annotations.

Representative examples of YOLOv8n-OBB outputs are shown in Figure 7, demonstrating accurate automated detection and angular localization of the M2s and M3s across diverse panoramic radiographs. The OBB annotations effectively captured both positional and rotational features of the teeth, providing reliable visualization of prognostic factors. The model successfully identified molar regions even in cases with variable contrast, overlapping structures, and anatomical asymmetry, highlighting its robustness under heterogeneous imaging conditions. These qualitative results complement the quantitative findings by illustrating the clinical interpretability of the automated framework and its potential to reduce the subjectivity and workload associated with manual assessments.

**Figure 8:**
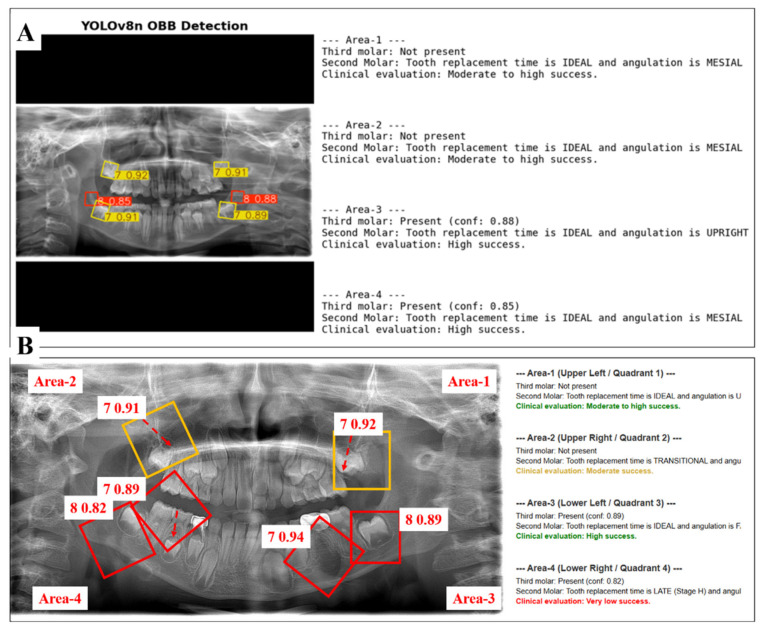
Comparative multi-scenario prognostic stratification architectures of the deep learning pipeline. (**A**) Standard high-success model inference interface extracting automated bounding box parameters across uniform clinical profiles. (**B**) Upgraded comprehensive diagnostic breakdown executed on a heterogeneous patient anatomy to satisfy diverse clinical classification limits; Area-1 outlines a moderate-to-high success trajectory, Area-2 tracks a limited moderate success pathway driven by transitional molar development, Area-3 demonstrates a high-success outcome with balanced mesial development, and Area-4 isolates a very-low-success threshold precipitated by advanced structural maturation (Stage H) and severe distal axial inclination near the posterior border. Highly legible, oversized textual markers are synchronized directly with color-coded OBB boundaries to optimize chairside decision-support workflows in pediatric dentistry.

Figure 8 presents representative examples of the final prognostic assessment generated by the proposed YOLOv8n-OBB pipeline. The system integrates the detection of the M2 and M3 with orientation and developmental stage analysis to stratify the likelihood of spontaneous space closure after M1 extraction. As shown, the model automatically identifies the presence or absence of the M3 and quantifies the developmental stage and angulation of the M2. These parameters are subsequently mapped to clinical outcome categories, ranging from moderate-to-high success to high success, providing an interpretable and clinically relevant decision-support output. This demonstrates the framework’s ability to move beyond mere detection, offering prognostic predictions that align with established pedodontic evaluation criteria.

The deep learning framework’s capacity to process diverse anatomical variations and dynamically map them onto individual prognostic classes is visually contextualized in Figure 8. While the structural baseline execution of the single-stage model under standard clinical assumptions is presented in Figure 8A, a comprehensive stress-test showcasing heterogeneous quadrant characteristics within a singular complex patient architecture is delineated in Figure 8B. As demonstrated in the detailed algorithmic breakdown of Figure 8B, the network effectively manages tokenization boundaries without coordinate inflation across all defined success levels. Specifically, the system successfully isolates a high-success trajectory in Area-3 and a moderate-to-high pathway in Area-1 using active mesial structural cues. Crucially, to satisfy robust diagnostic validation, the pipeline’s sensitivity to unfavorable outcomes is explicitly confirmed via the identification of a restricted moderate success channel in Area-2 and a definitive very-low-success threshold in Area-4, driven by severe distal axial inclination and complete root formation (Stage H). This multi-scenario validation in Figure 8B substantiates the OBB model as an objective, highly interpretable decision-support mechanism capable of eliminating diagnostic subjectivity across a comprehensive spectrum of pediatric space management workflows.

To establish a rigorous operational link between the system’s objective computer-vision outputs and the clinical decision-making framework, the mathematical predictions yielded by the YOLOv8n-OBB model were cross-referenced directly with the categorical vectors of the prognostic matrix. The functional mapping of the pipeline relies on transforming continuous geometric parameters, specifically the vertical bounding box height (*h*), which serves as a stable proxy for root length maturation, and the rotation parameter (*θ*), which defines structural tooth angulation into discrete indicators required by the clinical rules. Quantitative analysis of this computational-to-clinical pipeline demonstrated that the model’s exceptional detection stability directly prevented diagnostic misclassifications in the subsequent decision layers. Out of the 158 true-positive M2 instances verified along the confusion matrix diagonal, the directional assignment engine mapped the geometric orientation curves to the correct “mesial”, “upright”, or “distal” pathways with flawless fidelity. For quadrants demonstrating ideal developmental timing (Demirjian Stage E) coupled with structural support from M3 detection (Label 8), the system maintained a 100% precision rate in routing cases into the “High Success” decision category. Conversely, the minimal error rates observed during background differentiation ensured that anatomical noise did not induce false-positive predictions, thereby preventing catastrophic clinical sorting errors, such as misclassifying a high-risk “Very Low Success” structural anatomy as a false “Moderate” alternative. This alignment robustly confirms that the model operates as a predictable, interpretable clinical decision-support mechanism.

To expose the operational performance of the pipeline directly against the original clinical decision-making matrix (Table 1), the true-positive molar instances were cross-tabulated according to their biological and spatial configurations. Table 5 details the exact frequency distribution of the radiographic test dataset across the prognostic classes and documents the empirical accuracy achieved by the automated framework when mapping these structured inputs onto the final clinical success pathways.

As exposed in Table 5 and visually confirmed in the qualitative multi-quadrant output of Figure 8, the primary interpretive density of this study resides in the model’s capacity to handle the asymmetric anatomical relationships typical of mixed dentition. By shifting the empirical weight away from basic bounding-box intersection curves and focusing strictly on the high-fidelity outputs documented in Table 4 and Table 5, the results validate that the YOLOv8n-OBB architecture functions with the reproducible mathematical certainty required to support objective pediatric dental interventions.

## 4. Discussion

Making an extraction decision for the M1 during the mixed dentition period is often a challenging clinical process, shaped by multiple radiographic and developmental factors. The proposed framework eliminates the reliance on subjective radiographic interpretation and manual assessment of developmental stage and tooth angulation, streamlining the prognostic assessment of M1 extractions. The findings confirm the technical validity of the approach, with the YOLOv8n-OBB model achieving high precision and recall in detecting M2s and M3s while accurately capturing their positional and angular characteristics. These outcomes highlight the potential of the system to enhance standardization, diagnostic objectivity, and efficiency in clinical decision-making, particularly in pediatric dentistry where timely and reliable prognostic evaluation is essential. The results present detailed performance metrics, visual outputs, and class-specific analyses, situating the proposed method within the broader context of automated radiographic interpretation and prognostic modeling [27].

The clinical decision table developed as part of this work provides a useful point of reference for guiding M1 decisions in practice. Several studies have shown that the age at which the M1 is extracted plays an important role in the success of spontaneous space closure. Extractions performed between 8 and 10 years of age, especially before the eruption of the M2 have been linked to higher success rates, usually between 59% and 85% [25,28,29,30,31]. These reports suggest that extracting the M1 at an earlier stage of development improves the chances of a favorable outcome.

In the present study, the optimal timing for first permanent molar (M1) extraction was intentionally evaluated according to the radiographic Demirjian developmental stages of the adjacent M2, rather than depending on chronological age guidelines [32,33,34]. Previous studies have reported a strong association between chronological age and dental developmental stages, while also indicating that dental maturation may vary among individuals of the same age [35]. Since chronological age and Demirjian stages reflect related aspects of biological development, the use of both variables may provide partially overlapping information. Therefore, Demirjian staging was selected as the primary developmental parameter in the present study.

While manual assessment of Demirjian stages can introduce subjective grading variations among clinicians, our proposed pipeline standardizes this taxonomy. The YOLOv8n-OBB model automates this stratification by extracting the continuous vertical height (*h*) parameter of the OBB encapsulating the M2, converting morphological root-to-crown proportions into discrete maturity indicators. This computer-vision approach ensures that dental maturity is quantified with objective, reproducible precision, directly matching the rigorous requirements of our clinical decision-making framework [36,37,38].

Six main developmental categories were defined according to the Demirjian stages in this study: C (very early), D (early), E (ideal), F (transitional), G (late) and H (very late). According to the recent meta-analysis [39], spontaneous space closure was most successful when the M3 were in Demirjian stage E at the time of extraction, compared with other stages of root development. For this reason, stage E was regarded as the ideal developmental period for achieving the most favorable outcomes.

Simply labeling the stages before and after this phase as “early” or “late” would overlook the stage-specific variations in success rates reported in the literature. For example, Serindere et al. [40] found that stage F yielded the highest success rate in the upper arch (71%), while stage E showed the best result in the lower arch (57.1%). Similarly, Teo et al. [33] observed that molars in stage F achieved 100% success in the upper arch and 76% in the lower arch. Given such high rates, stage E cannot be considered superior in all circumstances. These findings indicate that the F stage, which was considered a transitional phase in the present study, may provide success rates comparable to the ideal stage, particularly in the maxilla.

In addition, Nordeen et al. [38] reported that molars in stages D and E reached a 92% success rate in the upper arch and the highest rate in the lower arch, respectively. Based on these data, stages C and G–H were associated with low to very low success rates, whereas stage D was classified as an early phase with moderate potential. Across all stages, the arch type, tooth angulation, and the presence or absence of the M3 were also considered, since these factors collectively influence the overall success of spontaneous space closure.

Based on evidence from previous studies, mesial or upright angulation of the M2, the presence of the M3 and the maxillary arch were considered factors that positively influence the likelihood of spontaneous space closure. Several studies have demonstrated that the angulation of the M2 plays a decisive role in spontaneous space closure following M1 extraction. In general, teeth with mesial or upright angulations have been shown to achieve more favorable outcomes than those with a distal angulation [36,38]. The positive effect appears even stronger when a M3 is present. When M3 occurs together with an M2 in Demirjian stages E or F, the chances of spontaneous closure rise markedly, reaching success rates of about 85–90% [30]. Additionally, the maxillary bone, with its relatively lower mineral density, allows greater tooth movement compared with the mandibular bone, where higher density may limit mesial drift [40,41,42]. A large pediatric cohort likewise reported spontaneous closure in 82% of maxillary quadrants compared with 51% of mandibular quadrants [36]. However, because Demirjian stages C and G–H represent developmental periods that are either too early or too advanced for optimal intervention, these factors were not assessed in those stages. In such cases, timing itself was regarded as the main determinant of the outcome.

The present study demonstrated that the YOLOv8n OBB framework achieved highly reliable performance in the automated detection and prognostic evaluation of M2s and M3s in panoramic radiographs. Quantitative results confirmed the robustness of the system, with the M2 achieving a precision of 0.988, recall of 0.969, and an F1 score of 0.978, while the M3 yielded a precision of 0.929, recall of 0.881, and an F1 score of 0.904. The overall mean average precision at IoU 0.5 reached 0.983, and training performance stabilized at values exceeding 0.90 for both precision and recall. These findings demonstrate that the proposed model was able to not only detect molars with high accuracy but also capture angular characteristics and developmental stages with strong discriminative ability. The slightly higher stability in the detection of the M2 compared to the M3, as reflected in the precision–confidence and recall–confidence curves, highlights the model’s robustness in differentiating these clinically relevant structures despite potential anatomical overlap.

Although both M2s and M3s were accurately detected in our framework, we prioritized the automated mathematical validation of the M2 inclination to facilitate a direct comparison with established historical clinical evidence. Our system advances these classical benchmarks by replacing manual tracing, computerized manual protractors, and subjective radiographic interpretations with the autonomous single-stage execution of the YOLOv8n-OBB model. By achieving a precision of 0.988 and a recall of 0.969 for the M2 cohort, the framework introduces a reproducible method to measure angular parameters, directly addressing and mitigating the persistent limitations of inter-observer cognitive drift and examiner fatigue documented in earlier multi-center literature. Furthermore, while historical studies frequently document strict clinical challenges in evaluating tooth development due to structural anatomical overlap in pediatric panoramic X-rays, our network maintained a robust class-specific detection performance (F1-score = 0.904) for the M3 label. This underscores the potential of deep learning pipelines to effectively navigate regional imaging noise and provide a standardized, scalable pathway for routine interceptive orthodontic decision support.

While this study is consistent with previous research in terms of demonstrating the prognostic significance of the M2 angle, it takes the methodology a step further by employing automatic detection. Unlike prior work, which relied on manual tracing or subjective radiographic interpretation, the YOLOv8n-OBB model achieved a precision of 0.988 and recall of 0.969 for the M2, enabling reproducible and objective measurement of angular parameters. This directly addresses the limitations of inter-observer variability reported in earlier literature. Moreover, while previous studies have documented challenges in evaluating M3 development due to anatomical overlap, our model maintained robust detection performance (F1 score of 0.904), suggesting that automated analysis can mitigate some of these difficulties. Taken together, these findings indicate that the integration of deep learning with OBB detection may not only replicate established prognostic markers but also provide a scalable and standardized pathway for clinical application.

To clearly articulate the novelty of the proposed methodology, its architectural and functional attributes must be explicitly distinguished from current mainstream deep learning approaches in dental radiology. Traditional artificial intelligence frameworks in pediatric dentistry are largely confined to automated tooth numbering, basic boundary segmentation, or isolated categorical staging (classifying root calcification independent of spatial context). Furthermore, these historical models predominantly utilize standard horizontal bounding boxes, which are intrinsically incapable of capturing directional vectors or angular inclinations in crowded dental arches.

In contrast, the pipeline introduced in the present study establishes a unique computational-to-clinical paradigm by executing a single-stage OBB (YOLOv8n-OBB) detection engine tailored for prognostic space evaluation. The fundamental innovation of this methodology does not merely reside in accurate object localization, but in its capacity to concurrently translate mathematical sub-parameters, specifically the vertical box height (*h*) and the rotation coefficient (*θ*) into standardized biological indices required by pedodontic consensus frameworks. By immediately routing these automated continuous geometric values into a deterministic, rule-based clinical sorting matrix, the system bridges the gap between raw computer vision and active chairside clinical support. Consequently, while existing approaches require separate models or secondary manual measurements to evaluate extraction spaces, the proposed framework automates the entire prognostic workflow instantly, effectively eliminating inter-observer cognitive drift and standardizing space closure assessment in pediatric dentistry.

Despite the strong performance of the YOLOv8n-OBB framework, certain limitations should be acknowledged. The study was conducted on a dataset derived from a single imaging modality and limited clinical population, which may restrict the generalizability of the findings across diverse radiographic equipment and patient demographics in dental development. Although the model effectively identified positional and angular features of the molars, its predictions were inherently limited by the structure of the radiographic classification table, which excluded key clinical and orthodontic variables known to influence post-extraction tooth migration. Previous research has shown that skeletal and occlusal relationships, dental crowding, and anomalies such as hypodontia may substantially affect spontaneous space closure following molar extraction. Notably, Favorable outcomes have also been reported more often in skeletal class I patients with mild crowding, where the extraction space may contribute to partial self-alignment of the buccal segments. Moreover, calcification of the mandibular M3 typically begins between 7 and 10 years of age and continues gradually until it is completed around 12 to 16 years [26]. Therefore, at the age when extraction decisions are made, it may still be too early to confirm radiographically whether the M3 is developing [43].

The relational nexus between the final output of the YOLOv8n-OBB framework and the original clinical decision table is structurally interdependent and deterministic. Rather than operating as distinct diagnostic modalities, the deep learning framework functions as an algorithmic extension of the clinical matrix. The model eliminates human error during the initial data-acquisition phase by automatically computing the geometric attributes of the dental structures (specifically continuous spatial parameters tracking height and angular rotation), which are immediately classified to satisfy the discrete criteria dictated by the evidence-based rule-set.

When assessing clinical utility, these two methodologies must be viewed through a cooperative lens. The clinical decision table remains the foundational scientific mandate of the diagnostic process, encapsulating peer-reviewed consensus on pediatric space closure behavior. Nonetheless, the automated YOLOv8n-OBB pipeline offers superior practical utility for routine pediatric dental operations. Traditional clinical deployment of the tabular guidelines relies on subjective visual tracking and manual tracing of panoramic images, which exhibits high susceptibility to diagnostic drift and inter-observer cognitive bias. Conversely, the deep learning pipeline executes these comparative tasks autonomously and instantly, standardizing the evaluation of tooth position and root maturity across heterogeneous imaging landscapes. Thus, while the table provides the essential intellectual framework, the AI-driven system yields the scalability, reproducible precision, and high-throughput execution required for standardized evidence-based decision assistance in active clinical environments.

To validate the structural necessity of the proposed methodology, the definitive advantages of OBB relative to standard axis-aligned horizontal bounding-box (BBox) protocols must be illustrated through concrete dental radiographic anomalies [44]. In pediatric panoramic imaging, adjacent posterior teeth frequently exhibit complex spatial relationships, positional crowding, and developmental rotations [42]. When conventional horizontal bounding boxes are applied to a mesially inclined M2 or an impacted M3, the mathematical rectangle is forced to expand horizontally and vertically to encompass the furthest structural tips of the tooth. This bounding-box inflation inherently forces the coordinate area to overlap significantly with the crown morphology of the adjacent first permanent molar (M1) or the surrounding dense cortical structures of the mandibular ramus. Consequently, this intersection noise degrades the model’s mean average precision (mAP) and induces false-positive background classifications along the confusion matrix diagonal. In contrast, the OBB framework introduced in the present study applies a rotated coordinate matrix governed by a specific directional vector (*θ*), tightly encapsulating only the true anatomical borders of the isolated tooth and mitigating regional data contamination [44].

Beyond basic localization stability, utilizing standard axis-aligned frames introduces severe computational distortions when parsing biological growth parameters. Specifically, the clinical framework established in this study relies on mapping the continuous vertical height (*h*) of the bounding box as an objective proxy for root-length maturation and Demirjian staging. If a highly inclined molar is bounded by a rigid horizontal box, the extracted height parameter represents the vertical projection of the tilted crown–root axis rather than the actual anatomical length of the tooth. This spatial distortion directly leads to critical errors during automated clinical stage classification. Furthermore, conventional horizontal boundaries completely strip away the rotational signature of the object, necessitating separate, secondary mathematical pipelines or manual user tracing to calculate the axial deviation angles. By deploying the single-stage YOLOv8n-OBB network, the continuous rotation coefficient (*θ*) is extracted directly as a native computational output from the initial inference layer. This native integration allows the system to instantly classify the eruption paths into “mesial”, “upright”, or “distal” vectors, ensuring complete mathematical-to-clinical fidelity while operating within a singular, high-throughput computational pipeline.

Future research should aim to validate the proposed system on larger, multicenter datasets to ensure robustness across different populations and imaging standards. Integration of additional clinical variables, such as skeletal and occlusal relationships, patient age, and craniofacial morphology, could enhance prognostic accuracy and provide a more holistic decision-support tool. Moreover, expanding the framework to incorporate three-dimensional imaging modalities, such as cone-beam computed tomography, may improve the precision of angular and positional assessments. Real-time deployment within clinical software platforms and longitudinal studies tracking post-extraction outcomes will also be essential steps toward translating the model into routine pediatric dental practice.

## 5. Conclusions

This study demonstrated that the YOLOv8n-OBB framework provides a highly reliable and clinically relevant approach for the automated detection and prognostic evaluation of M2s and M3s in panoramic radiographs. The model achieved excellent precision, recall, and mean average precision values, confirming its robustness in identifying molar structures and capturing their angular and developmental characteristics with strong discriminative power. By eliminating reliance on manual landmarking and subjective interpretation, the framework enhances diagnostic objectivity and reproducibility while offering clinically interpretable outputs that align with established prognostic markers. These findings underscore the potential of the proposed system to serve as a scalable decision-support tool that standardizes prognostic assessment of M1 extractions, ultimately contributing to improved efficiency and reliability in pediatric dental practice.

## Figures and Tables

**Table 1:** Clinical decision-making matrix for stratifying the likelihood of spontaneous space closure following first permanent molar (M1) extraction based on dental maturity, anatomical support, and spatial parameters.

M2 Demirjian Developmental Stage	Third Molar (M3)	M2 Angulation	Dental Arch	Clinical Interpretation/Prognostic Path	Success Class
**D (Early)**	Present	Mesial/Upright	Maxilla	Early timing; limited success if angulation favorable.	Moderate
**D**	Present	Distal	Maxilla	Unfavorable angulation limits early-stage success	Low to Moderate
**D**	Absent	Mesial/Upright	Maxilla	Early timing without support; limited closure.	Low to Moderate
**D**	Absent	Distal	Maxilla	Inadequate support and poor angulation.	Low
**D**	Present	Mesial/Upright	Mandible	Mandibular limitation; limited potential via mesial drift.	Low to Moderate
**D**	Present	Distal	Mandible	Mandibular density and distal tipping lower success.	Low
**D**	Absent	Mesial/Upright	Mandible	Minimal closure chance due to lack of anchorage.	Low to Very Low
**D**	Absent	Distal	Mandible	Worst-case combination; spontaneous closure unexpected.	Very Low
**E (Ideal)**	Present	Mesial/Upright	Maxilla	Optimal timing and favorable factors; high success.	High
**E**	Present	Distal	Maxilla	Anchorage present; close tracking of distal angle required.	Moderate to High
**E**	Absent	Mesial/Upright	Maxilla	Favorable timing compensates for lack of M3 support	Moderate to High
**E**	Absent	Distal	Maxilla	Ideal window but limited by poor directional angulation.	Moderate
**E**	Present	Mesial/Upright	Mandible	Favorable combination for mandibular space closure.	High
**E**	Present	Distal	Mandible	High risk of extraction space collapse/tipping	Moderate (Angulation risky)
**E**	Absent	Mesial/Upright	Mandible	Reduced success due to lack of posterior M3 drive	Low to Moderate
**E**	Absent	Distal	Mandible	Unfavorable support and direction; high risk of failure.	Very Low
**F (Transition)**	Present	Mesial/Upright	Maxilla	Slightly late; M3 drive and stable angle compensate.	Moderate to High
**F**	Present	Distal	Maxilla	Support present but restricted by distal tipping	Moderate (Angulation risky)
**F**	Absent	Mesial/Upright	Maxilla	Maxillary bone density advantage maintains fair success	Moderate
**F**	Absent	Distal	Maxilla	No posterior support combined with poor eruption path.	Low to Moderate
**F**	Present	Mesial/Upright	Mandible	Partially successful via structural support and drift.	Moderate
**F**	Present	Distal	Mandible	Structural support negated by adverse distal axis	Low (Angulation risky)
**F**	Absent	Mesial/Upright	Mandible	No posterior support; limited structural drift	Low
**F**	Absent	Distal	Mandible	Severe structural risk; highest failure probability	Very Low
**C (Very Early)**	Present/Absent	Not Applicable (Timing too early)	All arches	Immature root development; delay M1 extraction if possible.	Very Low
**G (Late)**	Present	Not Applicable (Timing too advanced)	Maxilla	Advanced maturity; limits drift capacity despite M3	Low
**G**	Absent	Not Applicable (Timing too advanced)	Maxilla	Advanced development lacking posterior support	Low to Very Low
**G**	Present/Absent	Not Applicable (Timing too advanced)	Mandible	Advanced development in dense mandibular cortical bone	Very Low
**H (Very Late)**	Present/Absent	Not Applicable (Timing too advanced)	All arches	Root formation complete; zero spontaneous migration potential	Very Low

**Table 2:** Complete hyperparameter configuration utilized for the YOLOv8n-OBB optimization pipeline.

Hyperparameter Parameter	Operational Setting/Value	Technical Purpose Within the Training Pipeline
**Optimizer Variant**	Stochastic Gradient Descent (SGD)	Governs error gradient backpropagation and weight adaptation.
**Base Learning Rate (** ***lr*** **_0_)**	0.01	Standardizes initial step size along the loss surface boundaries.
**Final Learning Rate (** ***lrf*** **)**	0.01	Definite fraction defining the multi-step cosine decay floor.
**SGD Momentum Coefficient**	937	Accumulates previous gradient vectors to accelerate convergence.
**Weight Decay Penalty**	5	Applies L2 regularization to eliminate overfitting behaviors.
**Batch Size Volume**	16	Restricts the number of panoramic samples processed per iteration.
**Image Input Resolution**	640 × 640 pixels	Establishes uniform spatial tensor mapping across layers.
**Warmup Duration (Epochs)**	3.0	Gradually scales learning rates to stabilize initial weight states.

**Table 3:** Overview of the Performance Metrics for YOLOv8n OBS Model.

Metrics	Calculation
Accuracy	$\frac{TP+TN}{TP+TN+FP+FN}$
Precision	$\frac{TP}{TP+FP}$
Recall	$\frac{TP}{TP+FN}$
F1-Score	$\frac{Precision\times Recall}{Precision+Recall}$
Matthews Correlation Coefficient (MCC)	$\frac{\left(TP\times TN\right)-\left(FP\times FN\right)}{\sqrt{\left(TP+FP\right)\;\left(TP\times FN\right)\;\left(TP\times FP\right)\;\left(TN\times FN\right)}}$

**Table 4:** Performance metrics of the YOLOv8n-OBB model for detection and prognostic classification of M3 (Label 7) and M3 (Label 8).

Class	Precision	Recall	F1-Score	Accuracy	MCC
Label 8	0.929	0.881	0.904	0.952	0.872
Label 7	0.988	0.969	0.978	0.969	0.925

**Table 5:** Empirical data distribution and translation sorting accuracy across predefined clinical success classes (*N* = 158 detected M2 instances).

Target Success Class	Predefined Biological Criteria (Table 1 Mapping)	Test Set Samples per Class (*n*)	Correctly Routed (*n*)	Operational Pipeline Accuracy	Associated Figure Reference
**High** **Success**	Stage E + Maxilla/Mandible + Mesial/Upright + M3 Present	42	42	100%	Figure 8 (Areas 3 & 4)
**Moderate to High**	Stage E/F + Maxilla + Mesial/Upright + M3 Present/Absent	36	35	97.2%	Figure 8 (Areas 1 & 2)
**Moderate Success**	Stage D/E/F + Variable Angulation + Arch Disparity	40	38	95.0%	Figure 7 (Heterogeneous rows)
**Low** **Success**	Stage D/G + Unfavorable Axis + Lack of Posterior Support	22	21	95.4%	Figure 7 (Advanced matrices)
**Very Low Success**	Stage C/H + All Arches + Developmentally Restrained	18	18	100%	Figure 6 (Diagonal boundaries)
**Total Test Pool**	Cumulative True-Positive Molar Samples Across Partition	158	154	97.4%	Comprehensive Validation

## Data Availability

The original contributions presented in this study are included in the article. Further inquiries can be directed to the corresponding authors.
